# Two-component signal transduction in *Corynebacterium glutamicum* and other corynebacteria: on the way towards stimuli and targets

**DOI:** 10.1007/s00253-012-4060-x

**Published:** 2012-04-28

**Authors:** Michael Bott, Melanie Brocker

**Affiliations:** Institut für Bio- und Geowissenschaften, IBG-1: Biotechnologie, Forschungszentrum Jülich, 52425 Jülich, Germany

**Keywords:** Histidine kinase, Response regulator, Sensors, Regulation, *C. diphtheriae*

## Abstract

In bacteria, adaptation to changing environmental conditions is often mediated by two-component signal transduction systems. In the prototypical case, a specific stimulus is sensed by a membrane-bound histidine kinase and triggers autophosphorylation of a histidine residue. Subsequently, the phosphoryl group is transferred to an aspartate residue of the cognate response regulator, which then becomes active and mediates a specific response, usually by activating and/or repressing a set of target genes. In this review, we summarize the current knowledge on two-component signal transduction in *Corynebacterium glutamicum*. This Gram-positive soil bacterium is used for the large-scale biotechnological production of amino acids and can also be applied for the synthesis of a wide variety of other products, such as organic acids, biofuels, or proteins. Therefore, *C. glutamicum* has become an important model organism in industrial biotechnology and in systems biology. The type strain ATCC 13032 possesses 13 two-component systems and the role of five has been elucidated in recent years. They are involved in citrate utilization (CitAB), osmoregulation and cell wall homeostasis (MtrAB), adaptation to phosphate starvation (PhoSR), adaptation to copper stress (CopSR), and heme homeostasis (HrrSA). As *C. glutamicum* does not only face changing conditions in its natural environment, but also during cultivation in industrial bioreactors of up to 500 m^3^ volume, adaptability can also be crucial for good performance in biotechnological production processes. Detailed knowledge on two-component signal transduction and regulatory networks therefore will contribute to both the application and the systemic understanding of *C. glutamicum* and related species.

**Electronic supplementary material:**

The online version of this article (doi:10.1007/s00253-012-4060-x) contains supplementary material, which is available to authorized users.

## Introduction

*Corynebacterium glutamicum* is a Gram-positive, facultative anaerobic, nonpathogenic soil bacterium which is used for the large-scale industrial production of the flavor enhancer l-glutamate (2.2 million tons in 2009) and the food additive l-lysine (1.5 million tons in 2011). Recent metabolic engineering studies have shown that *C. glutamicum* is also capable of producing a variety of other commercially interesting compounds, e.g. other l-amino acids (Wendisch et al. 2006a), d-amino acids (Stäbler et al. 2011), organic acids such as succinate (Okino et al. 2008; Litsanov et al. 2012a, b, c), diamines such as cadaverine (Mimitsuka et al. 2007) or putrescine (Schneider and Wendisch 2010), biofuels such as ethanol or isobutanol (Inui et al. 2004; Smith et al. 2010; Blombach et al. 2011), or proteins (Meissner et al. 2007). An overview of the product spectrum of *C. glutamicum* can be found in a recent review (Becker and Wittmann 2011). Due to its function as microbial cell factory, *C. glutamicum* has become a prominent model organism in industrial biotechnology and simultaneously for systems biology (Eggeling and Bott 2005; Wendisch et al. 2006b; Burkovski 2008). Another important aspect fostering research on *C. glutamicum* is its close phylogenetic relationship to important pathogens, in particular *Mycobacterium tuberculosis* (Ciccarelli et al. 2006). Both *Corynebacteriaceae* and *Mycobacteriaceae* belong to the suborder *Corynebacterineae* within the *Actinomycetales* (Stackebrandt et al. 1997).

Despite the industrial usage of *C. glutamicum* since the 1960s, studies on regulatory processes at the transcriptional and posttranscriptional level started only four decades later. LysG, the activator of the lysine exporter gene *lysE*, was the first transcriptional regulator to be reported (Bellmann et al. 2001). With the availability of genome sequences (Ikeda and Nakagawa 2003; Kalinowski et al. 2003; Yukawa et al. 2007), DNA microarrays for genome-wide expression analysis (Muffler et al. 2002; Hüser et al. 2003; Wendisch 2003), and proteomics (Hermann et al. 1998; Schaffer et al. 2001), research on regulation was boosted, particularly at the transcriptional level (for a review, see Schröder and Tauch 2010), but also at the posttranscriptional level (Bendt et al. 2003).

Protein phosphorylation is a key mechanism for the regulation of cellular activities at different levels. In *C. glutamicum*, four serine/threonine protein kinases (PknA, PknB, PknG, and PknL) and a single phosphoserine/phosphothreonine protein phosphatase have been annotated and were studied experimentally (Niebisch et al. 2006; Fiuza et al. 2008b; Schultz et al. 2009). A few target proteins of the kinases including phosphorylation sites have been identified, the first one being OdhI, a 15-kDa protein with a forkhead-associated domain. In its unphosphorylated state, OdhI inhibits the activity of the 2-oxoglutarate dehydrogenase complex by binding to the OdhA subunit, and this inhibition can be relieved by the PknG-catalyzed phosphorylation of OdhI at threonine-14 (Niebisch et al. 2006; Barthe et al. 2009; Krawczyk et al. 2010). This inhibition was shown to be crucial for glutamate production (Schultz et al. 2007). In addition, MurC (Fiuza et al. 2008a), FtsZ (Schultz et al. 2009), and RsmP (Fiuza et al. 2010), which are involved in peptidoglycan biosynthesis, cell division, and cell morphology, respectively, were identified as targets of serine/threonine protein kinases. Although phosphorylation of serine and threonine residues can also be involved in transcriptional regulation (Sharma et al. 2006a, b), histidine and aspartate phosphorylation by two-component signal transduction systems is the much more important and prevalent manner in bacteria.

## Basics of two-component signal transduction

**Fig. 1:**
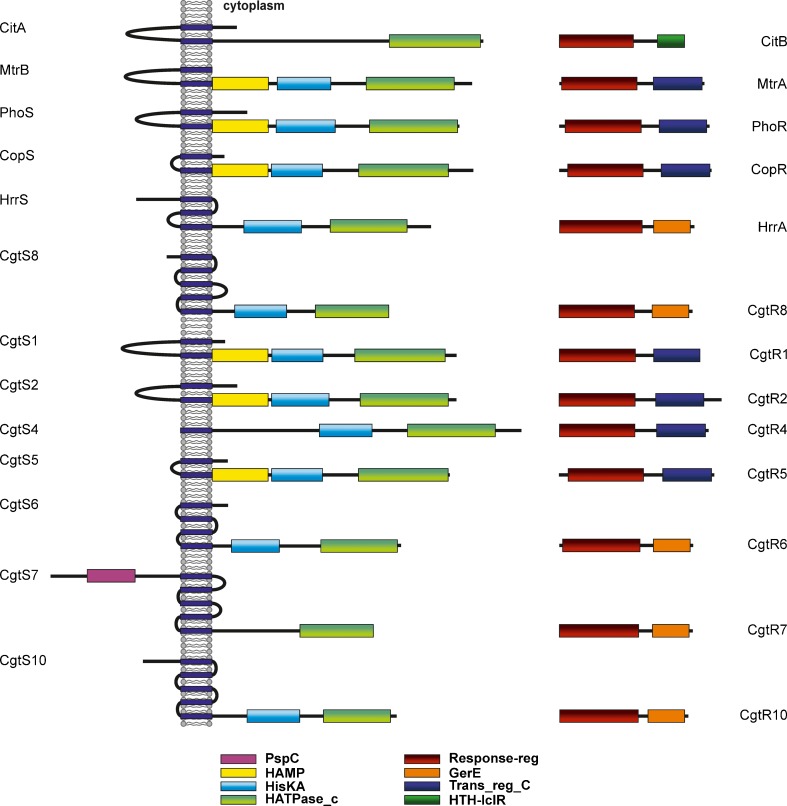
Schematic overview on the histidine kinases and their cognate response regulators of *C. glutamicum* ATCC 13032. The putative transmembrane helices were predicted by TopPred-II (von Heijne 1992; Claros and von Heijne 1994). Only candidates with a score above 1.2 were considered. The conserved domains and their location are indicated as predicted by PFAM (Punta et al. 2012)

Two-component systems (TCS) consist of a usually membrane-bound sensor kinase or histidine kinase (HK) and a response regulator (RR), which in most cases functions as transcriptional regulator. Both HKs and RRs are modular proteins (Fig. 1). Typical HKs are composed of a sensor domain, which is highly variable among different HKs, and a conserved cytoplasmic kinase core consisting of two distinct domains: a dimerization and histidine-phosphotransfer domain, designated HisKA domain in PFAM, and a C-terminal catalytic and ATP-binding (CA) domain, termed HATPase_c domain in the PFAM database (Punta et al. 2012). The HATPase domain binds ATP and catalyzes the transfer of the γ-phosphoryl group from ATP to the histidine residue, which is located within the HisKA domain. Several sequence motifs of the HATPase domain involved in ATP binding (G1, F, G2) are highly conserved. In many cases, additional domains such as HAMP domains are located between the N-terminal sensor domain and the C-terminal kinase core. Typical RRs are composed of a conserved N-terminal receiver domain (response_reg domain in PFAM), which contains the phosphorylatable aspartate residue, and a variable C-terminal effector or output domain. The HK responds to a certain stimulus by autophosphorylation of the conserved histidine residue in the HisKA domain and the phosphoryl group is subsequently transferred to the aspartate residue in the receiver domain of the RR in a reaction catalyzed by the RR. Phosphorylation activates (or in exceptional cases inhibits) the RR which then elicits a stimulus-specific response, usually the activation or repression of target genes (for reviews, see Stock et al. 1989, 2000; Bourret et al. 1991; Parkinson and Kofoid 1992; Mascher et al. 2006; Gao and Stock 2009). In this review, we summarize the experimental knowledge currently available for the TCS of the *C. glutamicum* type strain ATCC 13032 and we present an in silico analysis of TCS in *Corynebacterium* species for which complete genome sequences are available.

## Two-component systems in *C. glutamicum* ATCC 13032

The first *Corynebacterium* genome that was completely sequenced and analyzed with respect to TCS was the one of *C. glutamicum* ATCC 13032 (Ikeda and Nakagawa 2003; Kalinowski et al. 2003). Genes for 13 HKs and 13 RRs were identified, all forming HK–RR or RR–HK pairs (Kocan et al. 2006). No orphan HKs or RRs were found as in many other bacteria like *Escherichia coli* and *Bacillus subtilis* (Mizuno 1997; Fabret et al. 1999). Bioinformatic analyses revealed that the HKs as well as the RRs of *C. glutamicum* can be classified into three different classes, as shown in Tables 1 and 2 which also list the locus tags given in the different genome annotations. According to the classification scheme of Grebe and Stock, seven HKs belong to Histidine Protein Kinase subfamily HPK_1_, one to HPK_5_, and the remaining five to HPK_7_ (Grebe and Stock 1999). Classification of the RRs according to their output domains revealed a comparable distribution to the one of the sensor kinases: seven RRs belong to the OmpR family, one to the CitB family, and five to the LuxR family of RRs. Interestingly, all sensor kinases of HPK_1_ are paired with an OmpR-type response regulator, the HPK_5_-type sensor kinase is paired with the CitB-type RR, and all sensor kinases of HPK_7_ are paired with a LuxR-type RR (Fig. 1). All of the output domains of the *C. glutamicum* RRs contain DNA-binding motifs, suggesting that all of them function as transcriptional regulators (Kocan et al. 2006).

**Table 1:** Histidine kinases of *C. glutamicum* ATCC 13032

Histidine kinase	Locus tags	Class	Size (aa)	TMHs (aa position)	PFAM domains (aa position)	His ~ P site	Putative stimulus
CitA	Cg0089	5	551	27–47, 189–209	HisKA not identif.	355	Citrate
	NCgl0067				HATPase_c 435–548		
	Cgl0068						
MtrB	Cg0864	1	503	9–29, 175–195	HAMP 172–241	266	Unknown
	NCgl0722				HisKA 252–319		
	Cgl0755				HATPase_c 365–475		
PhoS (CgtS3)	Cg2887	1	485	44–64, 184–204	HAMP 185–255	276	Phosphate limitation
	NCgl2517				HisKA 266–330		
	Cgl2606				HATPase_c 373–484		
CopS	Cg3284	1	399	16–36, 66–86	HAMP 69–139	153	Copper
	NCgl2862				HisKA 143–207		
	Cgl2964				HATPase_c 253–366		
HrrS (CgtS11)	Cg3248	7	444	55–75, 90–110,	HisKA_3 208–281	217	Heme
	NCgl2835			148–168	HATPase_c 316–413		
	Cgl2937						
CgtS8 (ChrS)	Cg2201	7	377	13–33, 36–56,	HisKA_3 177–242	186	Heme
	NCgl1935			63–83, 105–125, 128–148	HATPase_c 279–371		
	Cgl2010						
CgtS1	Cg0331	1	489	14–34, 184–204	HAMP 185–254	268	Unknown
	NCgl0269				HisKA 258–322		
	Cgl0273				HATPase_c 362–475		
CgtS2	Cg0997	1	479	31–51, 171–191	HAMP 173–242	256	Unknown
	NCgl0840				HisKA 246–318		
	Cgl0875				HATPase_c 358–468		
SenX3 (CgtS4)	Cg0483	1	413	1–21	HisKA 156–222	166	Unknown
	NCgl0391				HATPase_c 267–378		
	Cgl0403						
CgtS5	Cg2948	1	372	20–40, 71–91	HAMP 73–143	157	Unknown
	NCgl2573				HisKA 147–211		
	Cgl2663				HATPase_c 257–370		
CgtS6	Cg3060	7	380	20–40, 57–77	HisKA_3 170–229	178	Unknown
	NCgl2667				HATPase_c 281–376		
	Cgl2763						
CgtS7	Cg0707	7	423	79–99, 118–138,	PspC 47–107	250	Unknown
	NCgl0585			142–162, 173–193, 199–219	HisKA_3 241–309		
	Cgl0611				HATPase_c 331–423		
CgtS10	Cg1083	7	419	48–68, 74–94,	HisKA_3 230–297	239	Unknown
	NCgl0911			109–129, 142–162, 165–185	HATPase_c 327–410		
	Cgl0948						

The locus tags are derived from the genome entries NC_006958, NC_003450, and BA000036, respectively. Classification was performed according to Grebe and Stock (1999). The transmembrane helices (TMHs, score above 1.2) were predicted by TopPred-II (Claros and von Heijne 1994). Domains and their position were calculated by PFAM (Punta et al. 2012). The phosphorylation sites (His ~ P) were predicted from sequence alignments

**Table 2:** Response regulators of *C. glutamicum* ATCC 13032

Response regulator	Locus tags	Class	Size (aa)	PFAM domains (aa position)	Asp ~ P site	DNA-binding site	No. of target genes	Auto-regula-tion
CitB	Cg0090	CitB	218	Response_reg 7–114	D57	n.d.	4	no
	NCgl0068			HTH_24 152–198				
	Cgl0069							
MtrA	Cg0862	OmpR	226	Response_reg 5–114	D53	8-bp tandem repeat	25	no
	NCgl0721			Trans_reg_C 146–222				
	Cgl0754							
PhoR (CgtR3)	Cg2888	OmpR	235	Response_reg 11–121	D59	8-bp tandem repeat	17	yes
	NCgl2518			Trans_reg_C 156–230				
	Cgl2607							
CopR	Cg3285	OmpR	240	Response_reg 15–124	D63	9-bp tandem repeat	9	yes
	NCgl2863			Trans_reg_C 160–237				
	Cgl2965							
HrrA (CgtR11)	Cg3247	LuxR	212	Response_reg 4–124	D54	n.d.	18	n.d.
	NCgl2834			GerE 149–206				
	Cgl2935							
CgtR8 (ChrA)	Cg2200	LuxR	210	Response_reg 4–122	D54	n.d.	n.d.	n.d.
	NCgl1934			GerE 147–204				
	Cgl2009							
CgtR1	Cg0330	OmpR	222	Response_reg 4–114	D52	n.d.	n.d.	n.d.
	NCgl0268			Trans_reg_C 150–221				
	Cgl0272							
CgtR2	Cg0996	OmpR	232	Response_reg 3–113	D51	n.d.	n.d.	n.d.
	NCgl0839			Trans_reg_C 152–227				
	Cgl0874							
RegX3 (CgtR4)	Cg0484	OmpR	232	Response_reg 4–113	D52	n.d.	n.d.	n.d.
	NCgl0392			Trans_reg_C 154–230				
	Cgl0404							
CgtR5	Cg2947	OmpR	241	Response_reg 16–125	D64	n.d.	n.d.	n.d.
	NCgl2572			Trans_reg_C 161–238				
	Cgl2662							
CgtR6	Cg3061	LuxR	206	Response_reg 7–119	D57	n.d.	n.d.	n.d.
	NCgl2668			GerE 147–204				
	Cgl2764							
CgtR7	Cg0709	LuxR	230	Response_reg 4–114	D52	n.d.	n.d.	n.d.
	NCgl0586			GerE 167–224				
	Cgl0612							
CgtR10	Cg1084	LuxR	203	Response_reg 4–118	D56	n.d.	n.d.	n.d.
	NCgl0912			GerE 140–197				
	Cgl0949							

The locus tags are derived from the genome entries NC_006958, NC_003450, and BA000036, respectively. Classification was performed according to the output domains. The domains and their position were predicted by PFAM (Punta et al. 2012). The aspartate phosphorylation sites (Asp ~ P) were predicted from sequence alignments

In order to test for the essentiality of the *C. glutamicum* ATCC 13032 TCS, a deletion study was performed which revealed that all TCS genes except for *regX3* (= *cgtR4*, cg0484) could be deleted (Kocan et al. 2006). Thus, of the 13 TCS only the SenX3-RegX3 system appears to be essential for growth.

## The CitA-CitB system: control of citrate utilization

The HK CitA and its cognate RR CitB of *C. glutamicum* belong to a family of TCS controlling the uptake and metabolism of citrate and dicarboxylates, the founding member being the citrate utilization (CitAB) TCS of *Klebsiella pneumoniae* (Bott et al. 1995). Citrate is a ubiquitous natural compound which can be utilized as a carbon and energy source by many bacterial species. Whereas anaerobic catabolism of citrate, which occurs for example in enteric bacteria (Bott 1997) and lactic acid bacteria (Bekal et al. 1998), requires a number of specific enzymes, in particular citrate lyase (Bott and Dimroth 1994), aerobic bacteria possessing a complete tricarboxylic acid cycle usually only require a citrate uptake system in order to be able to metabolize citrate.

*C. glutamicum* is able to grow aerobically in minimal medium with citrate as sole carbon and energy source (Polen et al. 2007; Brocker et al. 2009). When glucose is present in addition to citrate, both substrates are consumed simultaneously (Brocker et al. 2009), a feature typical for *C. glutamicum*. Global gene expression studies using DNA microarrays revealed that two putative citrate transport systems showed strongly increased expression in the presence of citrate, i.e. CitH (previously also named CitM or CitP) and TctABC (Polen et al. 2007). The former is a member of the citrate-Mg^2+^/H^+^/citrate-Ca^2+^/H^+^ symporter family (CitMHS), the latter belongs to the tripartite tricarboxylate transporter family (TTT). Expression of either *citH* or the *tctCBA* operon in *E. coli* enabled citrate utilization, confirming that both CitH and TctABC are functional citrate transporters (Brocker et al. 2009). Growth studies suggested that CitH is active with Ca^2+^ or Sr^2+^, but not with Mg^2+^, whereas TctABC is active with Mg^2+^ or Ca^2+^ but not with Sr^2+^. Evidence was obtained that 2 mM Ca^2+^ is sufficient to achieve maximal growth rates of *C. glutamicum* on citrate, whereas Mg^2+^ is required at 50-fold higher concentrations (Brocker et al. 2009). Either CitH alone or TctABC alone are sufficient for growth on citrate.

**Fig. 2:**
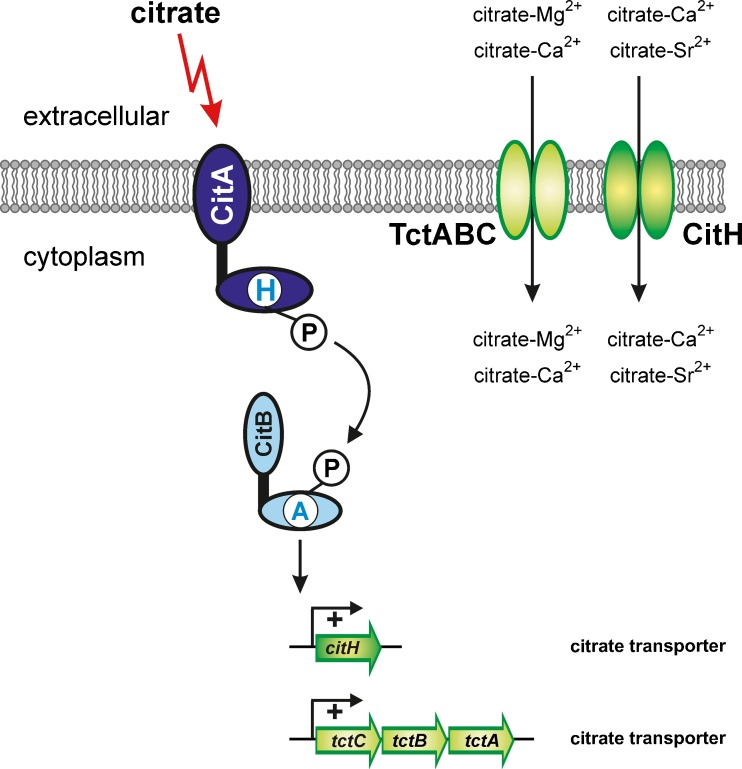
Control of citrate transport by the CitAB two-component system of *C. glutamicum*. The target genes shown in green are activated in the presence of extracellular citrate and encode two distinct citrate transport systems

The genes encoding the CitAB TCS of *C. glutamicum* are located immediately upstream of *citH* and in opposite direction. The HK CitA (58.6 kDa) contains two putative transmembrane helices that border an extracytoplasmic domain extending from residues 48–188. The RR CitB (23.4 kDa) is composed of the receiver domain and a DNA-binding domain belonging to the CitB family. A *C. glutamicum* mutant lacking the *citAB* genes was unable to grow with citrate as the sole carbon and energy source, but grew like wild type on glucose or pyruvate. The Cit^−^ phenotype could be abolished by transformation of the Δ*citAB* mutant with a *citAB* expression plasmid, confirming that the CitAB TCS is required for citrate utilization (Brocker et al. 2009). DNA microarray and primer extension experiments revealed that the citrate-inducible expression of both *citH* and *tctCAB* is strictly dependent on the CitAB TCS. Furthermore, the purified RR CitB was shown to bind to the promoter regions of *citH* and *tctCBA* (Brocker et al. 2009). The exact DNA-binding motif has not yet been identified. According to these results, CitA serves as a sensor for extracellular citrate and triggers the phosphorylation of CitB, which then activates the expression of the citrate transport genes *citH* and *tctCBA* (Fig. 2).

The question how citrate is sensed by CitA has not yet been answered experimentally for the protein of *C. glutamicum*, but for the HKs CitA from *K. pneumoniae* and *E. coli*. For *K. pneumoniae* it was shown that the isolated periplasmic domain CitAP binds citrate, presumably the dianionic form H-citrate^2−^, in a 1:1 stoichiometry with a *K*_d_ of 5 μM at pH 7 (Kaspar et al. 1999). In the case of *E. coli* CitA, the periplasmic domain bound citrate with a *K*_d_ of about 0.3 μM at pH 7 (Kaspar and Bott 2002). The crystal structure of *K. pneumoniae* CitAP in complex with citrate was the first one solved for a periplasmic domain of a HK and revealed a PAS-fold, a versatile ligand-binding structural motif. The groups responsible for citrate binding were identified as Thr-58, Arg-66, His-69, Ser-101, Leu-102, Lys-109, Ser124, and Arg-107. Four of these (R66, H69, R107, and K109) had been identified before as important for citrate binding by showing that their exchange to alanine increased the *K*_d_ 38- to >300-fold (Gerharz et al. 2003). In a subsequent study, structures of *K. pneumoniae* CitAP in the citrate-free and citrate-bound states were solved and their comparison showed that ligand binding causes a considerable contraction of the sensor domain (Sevvana et al. 2008). This contraction may represent the molecular switch that activates transmembrane signaling in the receptor, causing a piston-like movement of the second transmembrane helix towards the periplasm. In *C. glutamicum* CitA, all of the citrate-binding residues of *K. pneumoniae* CitA except Thr-58 and Ser-101 are conserved, suggesting that also the corynebacterial CitA protein directly senses the presence of citrate via its extracytoplasmic domain.

## The MtrB-MtrA system: osmoregulation and control of cell wall metabolism

The MtrAB TCS, which was the first one studied in *C. glutamicum*, is highly conserved in sequence and genomic organization in actinobacteria (Hoskisson and Hutchings 2006) and the RR MtrA was shown to be essential in *M. tuberculosis* (Zahrt and Deretic 2000). The RR MtrA of *C. glutamicum* is a 24.9 kDa protein with an OmpR-type DNA-binding output domain (Fig. 1). In contrast to *M. tuberculosis*, deletion of the *mtrA* gene alone and together with *mtrB* was possible in *C. glutamicum*. Δ*mtrAB* mutant cells exhibited a pleiotropic phenotype. The cells were elongated, segmented, and some showed irregular septum formation. In addition, they were more sensitive to penicillin and vancomycin, inhibitors of transpeptidases in cell wall synthesis, but more resistant to ethambutol, which interferes with the synthesis of the arabinogalactan moiety of the cell wall in *Corynebacterineae* (Belanger et al. 1996). These facts implied that the MtrAB TCS is somehow involved in cell wall homeostasis (Möker et al. 2004).

**Fig. 3:**
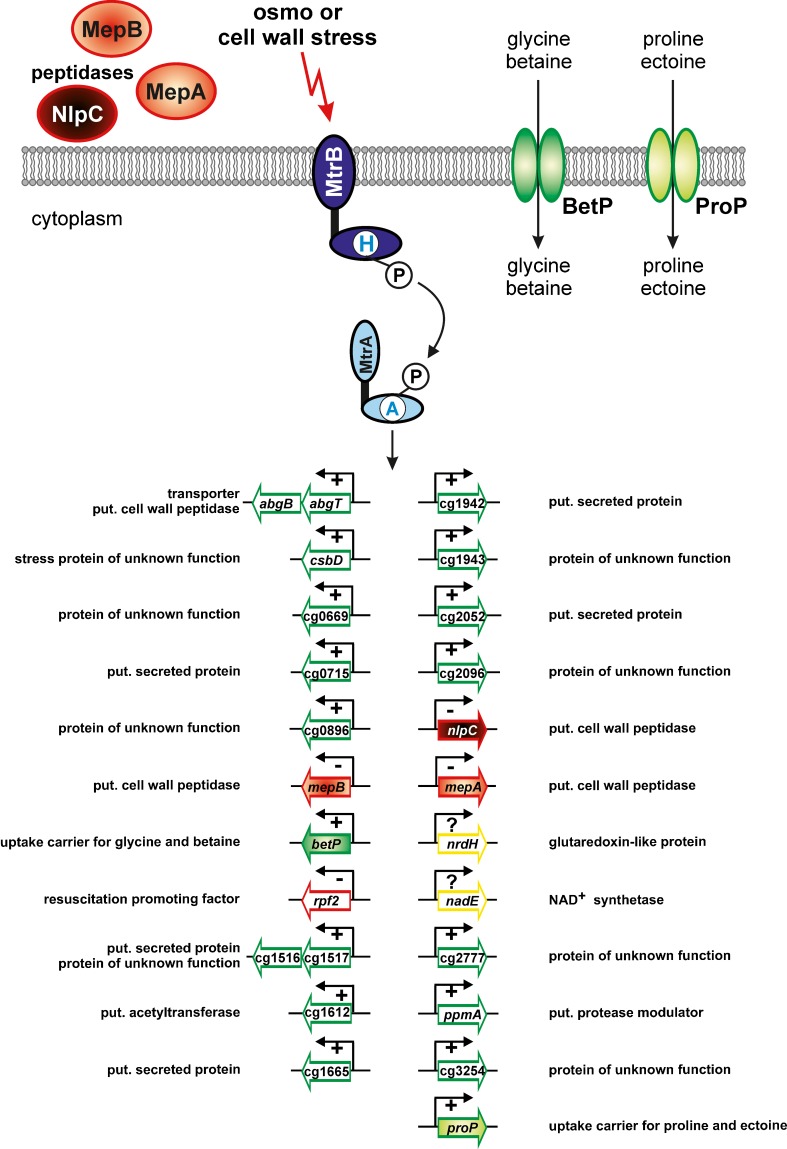
Regulon of the MtrAB two-component system of *C. glutamicum* showing its involvement in osmoregulation and cell wall metabolism. Genes shown in *red* are repressed and genes shown in *green* are activated by MtrA. Target proteins with known function are indicated. For genes highlighted in *yellow*, a binding of the response regulator MtrA upstream of these genes was observed, but the mRNA level of the genes was not altered in mutants lacking *mtrAB* or *mtrA*

DNA microarray analysis comparing the Δ*mtrA* or the Δ*mtrAB* mutant with the wild type combined with different types of MtrA-DNA-interaction studies (ChIP-chip analyses, DNA affinity chromatography, and electrophoretic mobility shift assays) revealed 22 MtrA target genes/operons, some being transcriptionally activated and some being transcriptionally repressed by MtrA (Fig. 3; Brocker and Bott 2006; Brocker et al. 2011). These contrary functions of activation and repression correlate with the position of the MtrA binding site in the promoter region of the corresponding target gene(s): it is located in the vicinity of the −10 region when MtrA acts as a repressor, whereas it is located upstream of the −35 region, when MtrA acts as an activator. In vitro phosphorylation of MtrA by phosphoramidate caused dimerization of the response regulator and enhanced its DNA-binding affinity, indicating that MtrA is activated by phosphorylation. The DNA-binding site of MtrA as determined by experimental work and bioinformatics was found to be a loosely conserved 8-bp direct repeat separated by a 3-bp linker (consensus sequence (A/G)TAACAATttn(A/G)TAACAAT), whose length is important for MtrA binding (Brocker et al. 2011). Direct repeats are often found as binding sites of OmpR-type regulators (Gupta et al. 2006; Hickey et al. 2011 and references therein). Based on structural studies with PhoB of *E. coli* it was proposed that upon phosphorylation OmpR-type RRs get activated by a mechanism in which the receiver domains form a twofold symmetric dimer while the DNA-binding domains bind to DNA with tandem symmetry (Bachhawat et al. 2005). The results obtained for MtrA of *C. glutamicum* are in accordance with such a mechanism.

Most of the MtrA target genes code for proteins of unknown function. Among the characterized target genes, *betP* and *proP* code for uptake carriers for the compatible solutes glycine betaine and proline or ectoine, respectively, which are involved in the response to hyperosmotic stress (Peter et al. 1998; Morbach and Krämer 2005). The genes *mepA*, *mepB*, and *nlpC* code for putative cell wall peptidases, *csbD* for a homolog of a protein belonging to the σ^B^-dependent general stress regulon in *B. subtilis* (Pragai and Harwood 2002), and *rpf2* for a resuscitation promoting factor (Hartmann et al. 2004). The phenotype of the Δ*mtrAB* mutant together with the function of the proteins encoded by the MtrA target genes indicated that the MtrAB system is involved in cell wall metabolism and the osmostress response. This conclusion is in agreement with experimental results on MtrA proteins of mycobacteria, which show high sequence identity to MtrA of *C. glutamicum* (e.g. 74 % to MtrA of *M. tuberculosis* [MtrA_*M.t.*_]). In *M. smegmatis*, downregulation of *mtrA* expression using an antisense mRNA technique resulted in elongated cells and an increased sensitivity to the antituberculosis drugs isoniazid and streptomycin (Li et al. 2010). In mycobacteria, several MtrA target genes have been described, such as the promoter regions of *dnaA* and *fbpB* coding for the initiator protein of DNA replication and the major secreted immunodominant antigen Ag85B, respectively, and the chromosomal origin of replication *oriC* (Fol et al. 2006; Li et al. 2010; Rajagopalan et al. 2010). MtrA_*M.t.*_ is constitutively expressed throughout growth in human macrophages (Haydel and Clark-Curtiss 2004) and MtrA can be detected in sera from TB patients (Singh et al. 2001) indicating that the MtrAB TCS is involved in pathogenesis in *M. tuberculosis*.

MtrB (54.7 kDa), the cognate HK of MtrA, contains two putative transmembrane helices which border an extracytoplasmatic domain of ~151 aa. To search for the stimulus sensed by MtrB, Strep-tagged MtrB was purified, reconstituted into proteoliposomes and its activities, autophosphorylation and phosphoryl group transfer to MtrA, were compared in the absence and presence of different stimuli (Möker et al. 2007a). Potassium ions were shown to stimulate MtrB activity, but this effect was also observed for DcuS of *E. coli*, a sensor kinase involved in recognition of C_4_ dicarboxylates. Therefore, K^+^ seems to have a general stimulation effect on HKs rather than being the stimulus of MtrB to sense hyperosmotic stress (Möker et al. 2007a). In subsequent studies, membrane shrinkage was excluded as being the specific stimulus for MtrB. Various compounds such as amino acids, sugars, and polyethylene glycols were shown to activate MtrB, presumably not by binding to a specific binding site, but by changing the hydration state of MtrB. As this activation was independent of the periplasmic loop and the HAMP domain (Fig. 1), the kinase domain was proposed to sense hypertonicity (Möker et al. 2007b).

Immediately downstream of *mtrA*–*mtrB*, the gene *lpqB* is located which encodes a lipoprotein of unknown function. In *M. tuberculosis*, it has been shown that the LpqB protein interacts with the extracellular domain of MtrB, thereby influencing MtrA phosphorylation and expression of the MtrA target gene *dnaA* (Nguyen et al. 2010). Hence, MtrAB together with LpqB seem to form a three-component system, as already suggested previously (Hoskisson and Hutchings 2006).

### The PhoS-PhoR system: coping with phosphate starvation

Phosphorus is one of the macroelements of all cells and makes up 1.5–2.1 % of the cell dry weight of *C. glutamicum* (Liebl 2005). Inorganic phosphate (P_i_) is the preferred phosphorus source of *C. glutamicum* and half-maximal growth rates are obtained at a P_i_ concentration of about 0.1 mM (Monod constant; Ishige et al. 2003). Besides P_i_, various other inorganic and organic phosphates can serve as phosphorus sources for *C. glutamicum* (Wendisch and Bott 2005; Wendisch and Bott 2008). When P_i_ is abundant, *C. glutamicum* accumulates up to 600 mM polyphosphate (Pallerla et al. 2005; Klauth et al. 2006) and various enzymes involved polyphosphate synthesis and degradation have been characterized (Lindner et al. 2007, 2009, 2010a, b).

When P_i_ becomes scarce, a genetic program is started and expression of a group of genes, called phosphate starvation inducible (psi) genes, increases (Ishige et al. 2003). From the kinetics of the transcriptional response, the following strategy to cope with P_i_ limitation was deduced. The first response after sensing Pi limitation is the increased expression of the *pstSCAB* operon, which encodes an ABC transporter for high affinity P_i_ uptake whose activity allows uptake of low residual P_i_ from the environment. Secondly, activated expression of *ugpAEBC* encoding an ABC transporter for glycerol 3-phosphate and *glpQ1* coding for a glyceryophosphoryl diester phosphodiesterase confers the ability to liberate glycerol 3-phosphate from lipids and to import this organophosphate into the cell. Simultaneously, increased expression of the *pctABCD* operon coding for an ABC transporter with yet unknown substrate specificity may allow the uptake of phosphorus-containing compounds. In a next phase, expression of a number of genes coding for secreted enzymes is increased allowing the mobilization of phosphate from nontransportable organophosphates. This group includes the *nucH*, *ushA*, and *phoC*. NucH is presumably a secreted nuclease for hydrolysis of extracellular DNA and RNA to desoxynucleotides and nucleotides. UshA was shown to be a secreted enzyme with UDP-sugar hydrolase and 5-nucleotidase activity, allowing access to phosphate in nucleotides (Rittmann et al. 2005). PhoC is proposed to function as a cell wall-associated phosphatase with unknown substrate specificity.

As TCS are obvious candidates for being involved in the regulation of the P_i_ starvation response, the set of 12 non-essential *C. glutamicum* TCS deletion mutants was screened for growth under P_i_ limitation. One of the mutants, strain Δ*phoRS* (originally named Δ*cgtRS3*), had a growth defect under P_i_ limitation, but not under P_i_ excess (Kocan et al. 2006). Interestingly, the *phoRS* genes were the only two-component genes whose expression was rapidly (within 10 min) and transiently induced after a shift from P_i_ excess to P_i_ starvation (Ishige et al. 2003). Both results suggested that the PhoRS TCS was involved in the adaptation to P_i_ limitation. Transcriptome comparisons and primer extension studies of the Δ*phoRS* mutant and the wild type demonstrated that none of the psi genes except *pstSCAB* was induced in the mutant within 60 min after a shift from P_i_ excess to P_i_ limitation. Activation of the *pstSCAB* genes was weaker in the Δ*phoRS* mutant than in the wild type (Kocan et al. 2006).

**Fig. 4:**
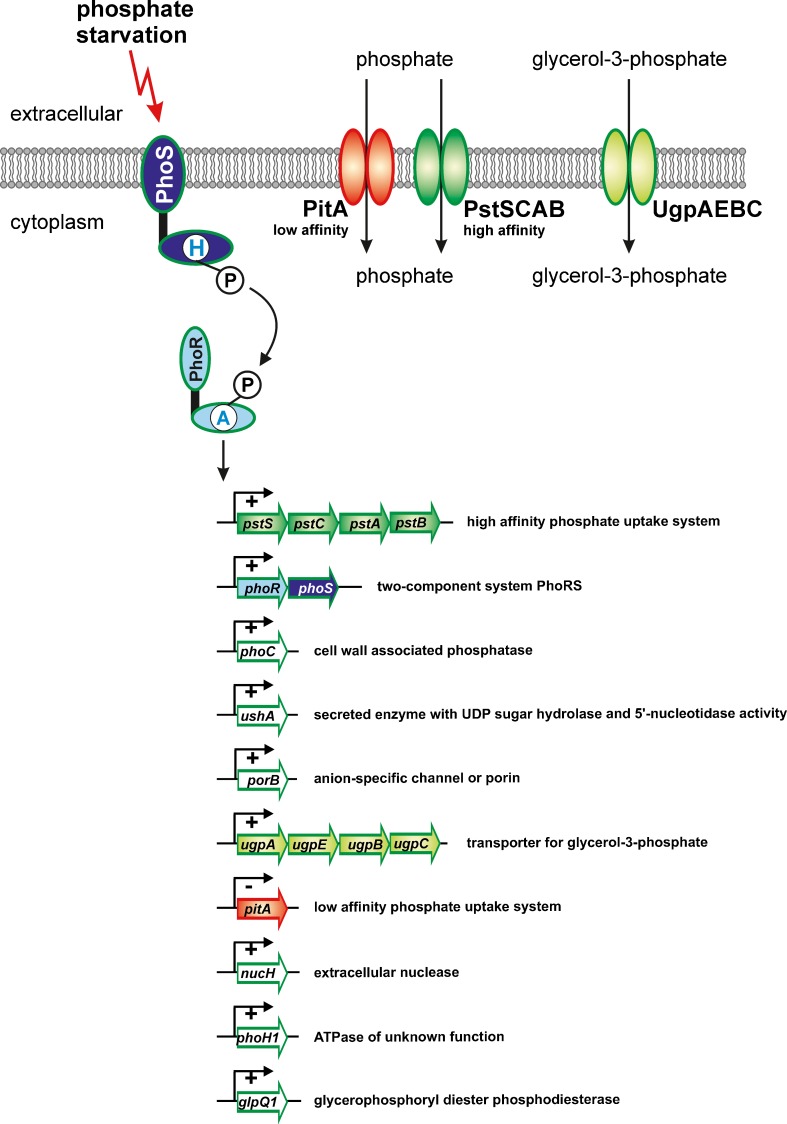
Role of the PhoRS two-component system of *C. glutamicum* in phosphate starvation. Genes shown in green are activated by PhoR, the *pitA* gene shown in *red* is repressed by PhoR. The function of some target proteins is indicated

The HK PhoS (52.4 kDa) contains two transmembrane helices delimiting an extracytoplasmic domain of about 120 amino acids and in the cytoplasm a HAMP domain followed by the HisKA and HATPase domains. The RR PhoR (26.4 kDa) is composed of an N-terminal receiver domain and a C-terminal output domain of the OmpR family (Fig. 1). In vitro studies revealed that the cytoplasmic part of PhoS showed constitutive autokinase activity and allowed rapid phosphorylation of PhoR. PhoR ~ P bound with different affinity to eight promoters of psi genes/operons, i.e. *pstSCAB*, *phoRS*, *phoC*, *ushA*, *ugpAEBC*, *nucH*, *phoH1*, and *glpQ1* (Fig. 4). In addition, PhoR ~ P also bound to the promoter of the porin gene *porB*, whose expression was reduced in the Δ*phoRS* mutant, and to the promoter of the *pitA* gene encoding a low-affinity secondary phosphate transporter (Schaaf and Bott 2007). Expression of *pitA* was reduced after a shift to P_i_ limitation and PhoR ~ P might act as a repressor of *pitA*. The affinity of unphosphorylated PhoR was about fivefold lower than that of PhoR ~ P, indicating that the latter is the active form of the protein (Schaaf and Bott 2007). The PhoR binding sites in the *pstSCAB* promoter and in the *phoRS* promoter were defined as 19-bp motifs composed of a loosely conserved 8-bp tandem repeat separated by a 3-bp linker, whose length is important for binding. The highest affinity was found for an artificial motif containing two perfect 8-bp tandem repeats: CCTGTGAAaatCCTGTGAA (Schaaf and Bott 2007). Again, this type of binding motif is in accord with that proposed for OmpR-type regulators (Bachhawat et al. 2005). The position of the binding motifs in the two promoters suggested different mechanisms of interactions with the RNA polymerase (Schaaf and Bott 2007).

The positive autoregulation of the *phoRS* genes, which were supported by reporter gene fusions, could be responsible for the successive expression of the psi genes. Whereas uninduced levels of PhoR ~ P could be sufficient to induce the high-affinity *pstSCAB* and *phoRS* promoters, elevated levels of PhoR ~ P might be required for induction of the lower affinity target promoters. Consequently, the PhoRS system may function as a rheostat rather than a simple switch.

The mechanism by which the PhoRS TCS senses phosphate limitation is still an open issue. Experiments in our laboratory with purified PhoS reconstituted into proteoliposomes argued against the idea that the environmental P_i_ concentration is directly sensed by PhoS. For the PhoR-PhoB TCS of *E. coli*, which performs a similar function in the phosphate starvation response as PhoRS in *C. glutamicum*, a model was proposed in which the phosphate ABC transporter PstSCAB is responsible for sensing the periplasmic P_i_ concentration and transfers this information to the sensor kinase PhoR, whereby also the PhoU protein plays a role (Hsieh and Wanner 2010). As a PhoU homolog is also present in *C. glutamicum* (encoded by cg2842), a similar mechanism as suggested for *E. coli* might be involved in P_i_ sensing by *C. glutamicum*.

## The CopS-CopR system: handling copper stress

Due to its ability to change between Cu(II) and Cu(I), copper serves as a redox cofactor for many enzymes, such as cytochrome *c* oxidases (Ridge et al. 2008). However, free copper ions can trigger the formation of reactive oxygen species and lead to sulfhydryl depletion. Therefore, high copper concentrations are toxic for cells. Most organisms have the ability to adapt to elevated copper concentrations by preventing the accumulation of free intracellular copper ions. Suitable strategies are for example the induction of copper exporters, of copper chaperons, or of multicopper oxidases to get rid of toxic intracellular copper levels, to sequester free copper ions, or to oxidize Cu(I) to the less toxic Cu(II), respectively (Osman and Cavet 2008).

Except for two putative multicopper oxidases, the only copper-dependent enzyme currently known in *C. glutamicum* is cytochrome *aa*_3_ oxidase, which forms a supercomplex with the cytochrome *bc*_1_ complex (Niebisch and Bott 2001, 2003; Bott and Niebisch 2003). As this supercomplex is critical for aerobic respiration and oxidative phosphorylation, copper is also required by *C. glutamicum*. On the other hand, copper concentrations of ≥50 μM inhibited growth of *C. glutamicum* (Schelder et al. 2011). DNA microarrays studies revealed that the genes encoding the HK CopS (cg3284) and the RR CopR (cg3285) as well as the up- and downstream genes (cg3286-cg3289 and cg3283-cg3281) showed strongly increased expression when cells were cultivated in the presence of 21 μM copper rather than at the routinely used 1 μM, indicating that this gene region is important for the adaptation to copper stress (Schelder et al. 2011).

**Fig. 5:**
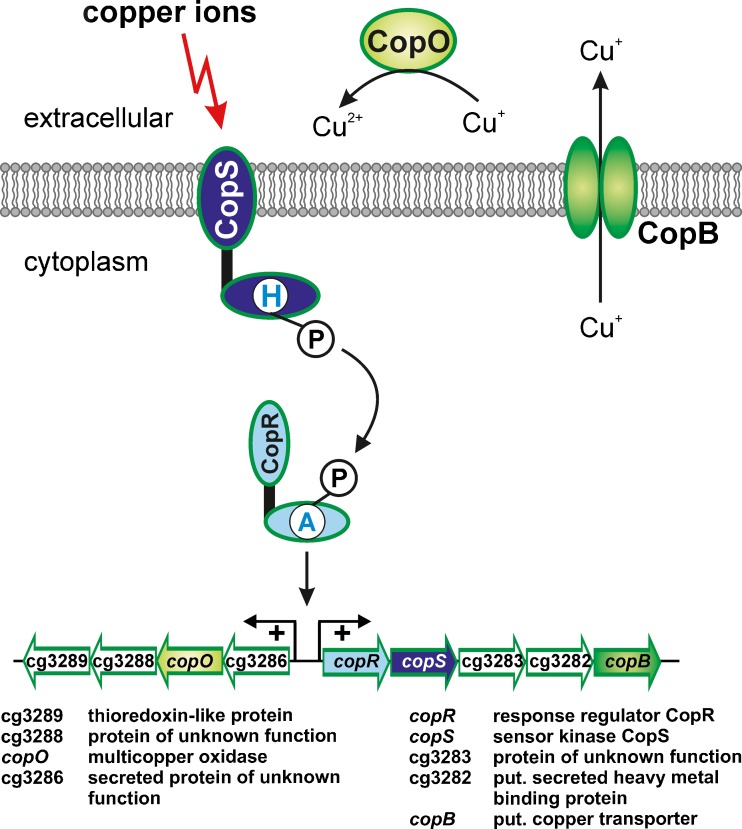
Response to copper stress by the CopRS two-component system of *C. glutamicum*. All genes displayed are activated by CopR in response to elevated copper concentrations

The relevance of the CopSR TCS for copper homeostasis was confirmed by the finding that a Δ*copSR* mutant showed an increased susceptibility to copper ions, but not to nickel, manganese, zinc, silver, cobalt, lead, or cadmium ions. This phenotype was reversed by plasmid-borne *copRS* expression in the Δ*copSR* mutant (Schelder et al. 2011). The HK CopS (43.0 kDa) presumably contains two transmembrane helices bordering a small extracytoplasmic region of about 30 amino acids, a HAMP domain and the characteristic HisKA and HATPase domains (Table 1 and Fig. 1). The RR CopR (26.7 kDa) is composed of a receiver domain and a DNA-binding domain of the OmpR family (Table 2 and Fig. 1). DNA microarray studies revealed no differences in gene expression between the Δ*copSR* mutant and the wild type when the strains were cultivated in glucose minimal medium with 1 μM copper, whereas 43 genes displayed a more than threefold altered mRNA level when the strains were grown in the presence of 21 μM copper. In particular, the genes cg3286-cg3289, which are located upstream of *copS* in reverse orientation showed 50- to 100-fold lower expression in the Δ*copSR* mutant, whereas expression of the three genes downstream of *copS*, cg3283-cg3281 was reduced only by a factor of about two. Binding studies with purified CopR uncovered a single binding site located in the intergenic region between *copR* and cg3286, which represents a 9-bp tandem repeat separated by 2-bp (TGAAGATTTnnTGAAGATTT). Phosphorylation by acetyl phosphate was shown to enhance the binding affinity of CopR to its DNA target about sixfold. Reporter gene assays indicated that CopR activates both the cg3286 and the *copR* promoter. According to these data, the CopSR system is activated by elevated copper levels and phosphorylated CopR activates expression of the cg3286-cg3287-cg3288-cg3289 genes and of the *copR*-*copS*-cg3283-cg3282-cg3281 genes (Fig. 5).

Some proteins encoded by these CopR target genes can obviously be linked to copper homeostasis: CopB (encoded by cg3281) is a putative copper export ATPase which exports copper out of the cytoplasm into the extracytoplasmatic space, where a putative multicopper oxidase (encoded by cg3287) can possibly oxidase Cu(I) to the less toxic and less membrane-permeable Cu(II). The function of the putative secreted copper-binding protein Cg3282 in copper homeostasis could be sequestration of excess copper ions or delivery of copper ions from CopB to the multicopper oxidase. The function of the other encoded proteins is still unclear.

Although there is clear evidence that CopS senses elevated copper concentrations, the mechanism of sensing has not yet been clarified. In principle, either CopS alone could be responsible for detection, or one or more additional proteins could be involved, such as a copper-binding protein or a copper transporter. For the copper-responsive HK CinS from *Pseudomonas putida*, a putative copper binding site has been identified which includes two histidine residues in the periplasmic loop (Quaranta et al. 2009). The periplasmic loop of CopS (LFHDHMLMTGREDPSLELFHAEQAYRDAN) also contains three histidine residues as well as two methionine residues, which might also be involved in direct copper binding as shown for other copper-binding proteins (Davis and O'Halloran 2008).

## The HrrS-HrrA system: control of heme homeostasis

Heme is a cofactor of various enzymes, in particular complexes of the respiratory chain, but can also serve as a source of iron. In *C. glutamicum*, prominent heme-containing enzymes are succinate dehydrogenase, also called succinate:menaquinone oxidoreductase (Kurokawa and Sakamoto 2005), the cytochrome *bc*_1_-*aa*_3_ supercomplex (Niebisch and Bott 2001, 2003; Sone et al. 2001), cytochrome *bd* oxidase (Kusumoto et al. 2000), respiratory nitrate reductase (Bott and Niebisch 2003), or catalase. Thus, both aerobic and anaerobic respiration of *C. glutamicum* are strictly dependent on heme (Bott and Niebisch 2003; Nishimura et al. 2007; Takeno et al. 2007) as well as the detoxification of reactive oxygen species generated by respiration. Heme biosynthesis in *C. glutamicum* occurs via the C5 pathway that uses glutamate as the substrate for the synthesis of δ-aminolevulinic acid (Bott and Niebisch 2003).

As heme is an iron-containing porphyrin, heme metabolism is intimately connected with iron metabolism. Similar to copper, iron is both essential as protein cofactor but also dangerous as ferreous iron catalyzes the formation of reactive oxygen species. Therefore, organisms have evolved sophisticated strategies to ensure sufficient iron supply, but to avoid high, toxic intracellular iron concentrations (Andrews et al. 2003). In *C. glutamicum*, DtxR has been identified as the master regulator of iron homeostasis (Brune et al. 2006; Wennerhold and Bott 2006; Frunzke and Bott 2008). Among the target genes repressed by DtxR under iron excess were several ones related to heme metabolism, i.e. a putative operon predicted to encode a secreted heme transport-associated protein (*htaA*, cg0466) and an ABC transporter for heme uptake (*hmuTUV*, cg0467-cg0468-cg0469), a putative operon predicted to encode to further secreted heme transport-associated proteins (*htaB*-*htaC*, cg0470-cg0471), another putative secreted heme transport-associated protein (*htaD*, cg3156), and the *hmuO* gene (cg2445) encoding heme oxygenase (Wennerhold and Bott 2006). The presence of genes related to heme import and degradation which are derepressed under iron limitation suggested that the non-pathogenic *C. glutamicum* can use heme as an iron source. In fact, non-toxic concentrations of hemin (2.5 μM) as sole iron source allowed comparable growth rates and cell yields as the same concentration of FeSO_4_ (Frunzke et al. 2011). Mutants lacking either the *hmu* operon (*htaA-hmuTUV*) or the *htaBC* operon showed a slight, but significant growth defect when hemin was supplied as iron source, whereas growth of a Δ*hmuO* mutant lacking heme oxygenase was strongly impaired. These phenotypes support the involvement of the corresponding proteins in heme uptake and degradation. Transcriptome studies revealed that all of the aforementioned genes related to heme metabolism and a few additional ones showed increased expression levels when heme was used as sole iron source (Frunzke et al. 2011).

The set of target genes repressed by DtxR under iron-sufficient conditions in *C. glutamicum* also includes genes for transcriptional regulators, in particular those for the AraC-type regulator RipA (Wennerhold et al. 2005) and for the RR CgtR11. The gene *cgtR11* (*hrrA*) is located downstream of *cgtS11* (*hrrS*) encoding the cognate HK, which however is not repressed by DtxR (Wennerhold and Bott 2006). Due to the high sequence identity of the CgtSR11 system of *C. glutamicum* to the HrrSA TCS of *C. diphtheriae* (Bibb et al. 2007), it was renamed accordingly. The *C. diphtheriae* HrrSA system was shown to be involved in the heme-dependent activation of *hmuO* and repression of *hemA*, encoding the heme biosynthesis enzyme glutamyl-tRNA reductase (Bibb et al. 2007). A Δ*hrrA* mutant of *C. glutamicum* showed a strong growth defect on agar plates containing hemin as sole iron source, suggesting that also the *C. glutamicum* HrrSA system plays a role in heme metabolism. Transcriptome comparisons of the Δ*hrrA* mutant and the wild type and in vitro studies with purified HrrS and HrrA protein led to the identification of six promoter regions to which HrrA binds and to the definition of the HrrA regulon (Frunzke et al. 2011). The DNA binding motif of HrrA, which is composed of a receiver domain and a LuxR-type DNA binding domain (Fig. 1), has not yet been determined.

**Fig. 6:**
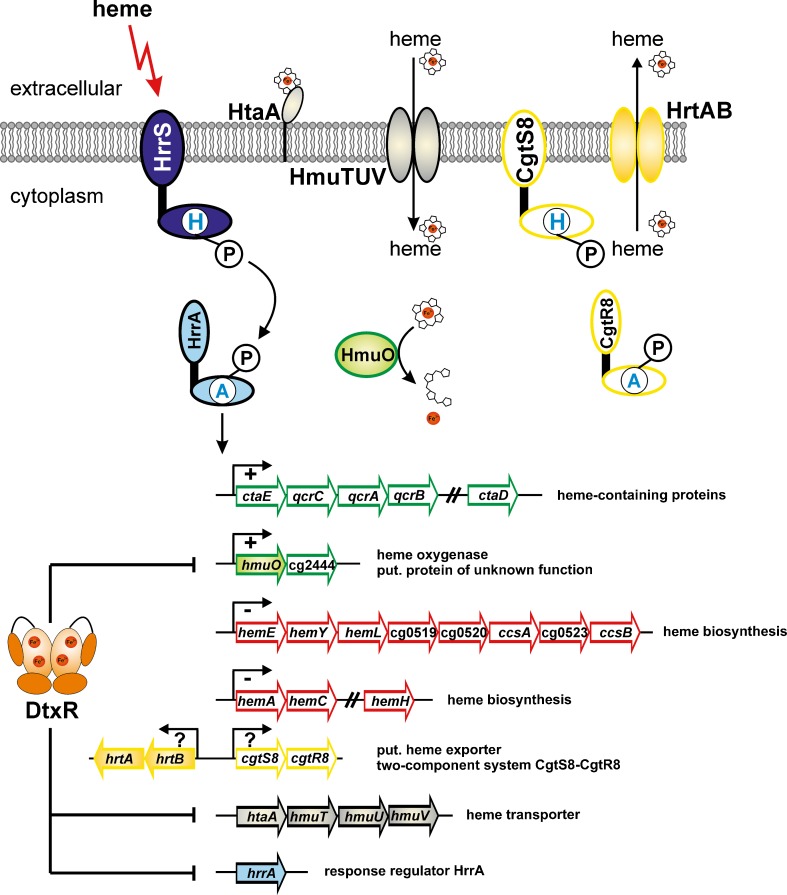
Control of heme homeostasis by the HrrSA two-component system of *C. glutamicum*. The genes shown in green are activated by HrrA, the genes encircled in red are repressed by HrrA. The genes shown in *black*, which encode an ABC transporter for heme uptake, are not regulated by HrrA. Rather, they are repressed under iron excess by DtxR, similar to some of the HrrA target genes. For genes highlighted in *yellow*, a binding of the response regulator HrrA upstream of these genes was observed. It is still unclear if binding of HrrA to this region leads to activation or repression of one or both of the divergently orientated operons. The CgtSR8 two-component system possibly activates expression of the *hrtBA* genes, which encode a putative heme exporter, in response to elevated heme concentrations. Note that alternative names exist for some of the genes involved in heme biosynthesis: *hemY*/*hemG*, cg0520/*ccsX*/*resA*, *ccsA*/*ccdA*, cg0523/*ccsB*/*resB*, *ccsB*/*ccsA*/*resC*

Like many other RR, HrrA functions both as an activator and as a repressor. The genes activated by HrrA ~ P code for heme oxygenase (*hmuO*), for subunit III of cytochrome *aa*_3_ oxidase and the three subunits of the cytochrome *bc*_1_ complex (*ctaE-qcrCAB* operon), and for subunit I of cytochrome *aa*_3_ oxidase (*ctaD*). The genes repressed by HtrA ~ P code for 11 proteins involved in heme biosynthesis and cytochrome *c* maturation (*hemE*-*hemY*-*hemL*-cg0519-*ccsX*-*ccdA*-*resB*-*resC*; *hemA*-*hemC*; *hemH*). Thus, when heme is available, HrrSA stimulates heme degradation and the synthesis of the heme-containing cytochrome *bc*_1_-*aa*_3_ supercomplex and at the same time reduces heme biosynthesis. It thus plays a key role in heme homeostasis, together with the master regulator DtxR (Fig. 6).

The HrrSA TCS is assumed to be activated by heme, but biochemical studies confirming this assumption are not yet available. The HK HrrS (Fig. 1) is predicted to contain three transmembrane helices that border two extracytoplasmic regions of about 54 and 38 amino acid residues and the conserved HisKA and HATPase domains (Kocan et al. 2006). Heme recognition could occur either at the periplasmic side or within the cytoplasmic membrane.

## The CgtS8-CgtR8 system: a second TCS possibly involved in heme homeostasis

Besides HrrSA, a second TCS of *C. glutamicum*, CgtSR8, might also be involved in the regulation of heme homeostasis. The CgtSR8 system shows high sequence identity to the ChrSA system of *C. diphtheriae*, which activates *hmuO* expression and represses *hemA* expression in a heme-dependent manner (Schmitt 1999; Bibb et al. 2005, 2007). More recently, also expression of the *hrtBA* genes of *C. diphtheriae*, which code for an ABC transporter conferring resistance to heme toxicity, was reported to be activated by ChrSA in this bacterium (Bibb and Schmitt 2010). Genes homologous to *hrtBA* (cg2202, cg2204) are also present in the *C. glutamicum* genome immediately upstream of *cgtSR8* in opposite orientation and expression of both genes was activated by heme (Frunzke et al. 2011). Furthermore, the RR HrrA was shown to bind to the *cgtS8-hrtB* intergenic region and expression of *cgtSR8* and *hrtBA* was increased two- and ten-fold, respectively, in the Δ*hrrA* mutant during growth with heme as sole iron source. These data suggest that CgtSR8 might have a similar function as ChrSA in *C. diphtheriae* and that HrrSA and CgtSR8 of *C. glutamicum*, which show sequence similarity to each other, are interrelated (Frunzke et al. 2011).

For ChrS of *C. diphtheriae*, experimental evidence was provided that it functions as heme sensor. The autophosphorylation of purified ChrS reconstituted into proteoliposomes was shown to be stimulated by 1 μM hemin, but not by other metalloporphyrins and iron. In addition, UV-spectra supported a direct interaction between ChrS and hemin (Ito et al. 2009). The N-terminal region of ChrS (and CgtS8) is predicted to contain five transmembrane helices and the deduced topology was supported by PhoA and LacZ fusions (Bibb and Schmitt 2010). Distinct amino acid substitutions (R34A, Y61F, R70A, D75N, F114N) in the N-terminal region of ChrS were found to inhibit or prevent heme-dependent activation of the *hrtB* promoter or to cause heme-independent, constitutive promoter activation (H21L). These data support a role of the N-terminal ChrS region in heme sensing and signal transfer to the kinase domain (Bibb and Schmitt 2010).

## Conservation of the *C. glutamicum* two-component systems in other species of corynebacteria

Except for the TCS of *C. glutamicum* ATCC 13032 described above and the HrrSA and ChrSA systems of *C. diphtheriae*, no other TCS of corynebacteria have been experimentally studied to our knowledge. However, a variety of genome sequences of *Corynebacterium* species were determined in recent years, which are of interest because of their pathogenicity, their role in cheese ripening, or in amino acid production. We performed an *in silico* analysis of these genomes to determine the conservation of the *C. glutamicum* TCS. The results of this analysis are summarized in Table 3 and Table S1, which lists the GI numbers of the orthologous proteins.

**Table 3:** Two-component signal transduction systems in *Corynebacterium* species

Two-component system	Presence in the indicated *Corynebacterium* strains^a^																					
	Cgl^b^	CglR^b^	Cau^b^	Cdi^b^	Cef^b^	Cje^b^	Ckr^b^	Cpt^b^	Cur^b^	Cul^b^	Cva^b^	Cac1	Cam	Cbo	Cge	Cgc1	Cli	Cma1	Cpg	Cre	Cst	Ctu
												Cac2				Cgc2		Cma2				
CitAB	+	+			+						+											
MtrBA	+	+	+	+	+	+	+	+	+	+	+	+	+	+	+	+	+	+	+	+	+	+
PhoSR	+	+	+	+	+	+	+	+	+	+	+	+		+	+	+	+	+	+	+	+	+
CopSR	+	+		+	+	+	+					+			+	+	+		+		+	+
HrrSA	+	+	+	+	+			+		+		+			+	+	+	+	+		+	+
CgtSR8	+	+		+		+		+	+				+				+			+		
CgtSR1	+	+			+												+	+				
CgtSR2	+	+	+	+	+	+		+	+	+			+		+	+		+	+	+	+	+
CgtSR4	+	+	+	+	+	+	+	+		+	+	+	+	+	+	+	+		+	+	+	+
CgtSR5	+	+																				
CgtSR6	+																					
CgtSR7	+	+^d^	+	+	+			+	+	+					+		+	+		+	+	
CgtSR10	+	+			+						+	+^c^									+	
cgR_2292		+																				
cgR_2299		+																				
cgR_0540/0541		+							+						+							

+, Genes encoding the sensor kinase and the response regulator are present

^a^Cgl, *Corynebacterium glutamicum* ATCC 13032 (Ikeda and Nakagawa 2003; Kalinowski et al. 2003); CglR, *Corynebacterium glutamicum* R (Yukawa et al. 2007); Cau, *Corynebacterium aurimucosum* ATCC 700975 (Trost et al. 2010a); Cdi, *Corynebacterium diphtheriae* NCTC-13129 (Cerdeno-Tarraga et al. 2003); Cef, *Corynebacterium efficiens* YS-314 (Nishio et al. 2003); Cje, *Corynebacterium jeikeium* K411 (Tauch et al. 2005); Ckr, *Corynebacterium kroppenstedtii* DSM 44385 (Tauch et al. 2008a); Cpt, *Corynebacterium pseudotuberculosis* FRC41 (Trost et al. 2010b); Cur, *Corynebacterium urealyticum* DSM 7109 (Tauch et al. 2008b); Cul, *Corynebacterium ulcerans* BR-AD22 (Trost et al. 2011); Cva, *Corynebacterium variabile* DSM 44702 (Schröder et al. 2011); Cac1, *Corynebacterium accolens* ATCC 49725; Cac2, *Corynebacterium accolens* ATCC 49726; Cam, *Corynebacterium amycolatum* SK46; Cbo, *Corynebacterium bovis* DSM 20582; Cge, *Corynebacterium genitalum* ATCC 33030; Cgc1, *Corynebacterium glucuronolytium* ATCC 51866; Cgc2, *Corynebacterium glucuronolytium* ATCC 51867; Cli; *Corynebacterium lipophiloflavum* DSM 44291; Cma1, *Corynebacterium matruchotii* ATCC 14266; Cma2, *Corynebacterium matruchotii* ATCC 33806; Cpg, *Corynebacterium pseudogenitalium* ATCC 33035; Cre, *Corynebacterium resistens* DSM 45100; Cst, *Corynebacterium striatum* ATCC 6940; Ctu, *Corynebacterium tuberculostearicum* SK141

^b^Completed and published genomes

^c^Only present in Cac2

^d^Only the gene encoding the response regulator is present (cgR_0730)

In *C. glutamicum* strain R, genes for 13 sensor kinases and 14 response regulators are found (Yukawa et al. 2007). Homologs of CgtSR6 and of CgtS7 are absent, while two TCS not present in strain ATCC 13032 are found in strain R (cgR_2292, cgR_2299, cgR_0540, cgR_0541). Thus, even in strains of the same species the presence of TCS can vary.

Considering their conservation in *Corynebacterium* strains with known genome sequence, the TCS can be divided into three groups. Group A contains three highly conserved TCS, namely MtrAB, PhoSR and CgtSR4 (SenX3/RegX3). They are present in all (MtrAB) or all except for one (PhoRS) or two (SenX3/RegX3) species analyzed here. It can be assumed that these TCS play important roles in the physiology of corynebacteria, which is supported in the case of MtrAB and PhoRS by the large regulons and in the case of CgtR4 by its essentiality in *C. glutamicum* ATCC 13032. Group B involves four TCS that are present in 12–18 of the 22 species analyzed here: CopSR, HrrSA, CgtSR2, and CgtSR7. The function of the latter two is not yet known. Finally group C contains nine TCS that are found in one to nine species. Of these, only the function of the CitAB and the ChrSA (CgtSR8) system is currently known.

## Concluding remarks

The work summarized above illustrates that significant progress was achieved in understanding the role of TCS in *C. glutamicum* since the genome sequence was published in 2003 and allowed the *in silico* identification of these signal transduction systems. Cellular functions were elucidated for five of the 13 TCS present in the type strain ATCC 13032 by using the following approaches: (1) comparison with TCS of known functions from other bacteria; (2) inspection of the genomic environment of the TCS genes; (3) search for phenotypes of deletion mutants lacking a particular TCS; and (4) identification of the target genes of the RRs. The five characterized systems include CitAB (citrate uptake), MtrAB (osmoregulation and cell wall homoeostasis), PhoSR (phosphate limitation), CopSR (copper stress), and HrrSA (heme homeostasis). The stimuli sensed by the corresponding HK are predicted to be citrate (CitA), phosphate limitation (PhoS), copper ions (CopS), and heme (HrrS); however, direct biochemical evidence for these predictions is not yet available. Further studies along the lines outlined above should allow to elucidate the roles of all 13 TCS of *C. glutamicum*. The resulting knowledge will contribute to a systemic understanding of this species and can be used for optimization of strains or process conditions used for industrial purposes.

## Electronic supplementary materials

Below is the link to the electronic supplementary material.

ESM 1 PDF 18.8 kb
