# Supplementary material for: Two-component signal transduction in Corynebacterium glutamicum and other corynebacteria: on the way towards stimuli and targets
Source: Appl Microbiol Biotechnol. 2012 Apr 28;94(5):1131–50. doi: 10.1007/s00253-012-4060-x (PMC3353115; doi:10.1007/s00253-012-4060-x)
Supplement: Supplementary file 1 — PDF 18.8 kb [file 253_2012_4060_MOESM1_ESM.pdf]

Supplementary material

Two-component signal transduction in *Corynebacterium glutamicum* and other corynebacteria: on the way towards stimuli and targets

Michael Bott and Melanie Brocker

Institut für Bio- und Geowissenschaften, IBG-1: Biotechnologie, Forschungszentrum Jülich, D-52425 Jülich

Table S1. Two-component signal transduction systems in *Corynebacterium* species. The proteins orthologous to the histidine kinases and response regulators present in *C. glutamicum* are listed by their GI numbers in NCBI.

Two-component system	GI number of the corresponding orthologous protein in the indicated *Corynebacterium* strain[a]																								
	Cgl[b]	CglR[b]	Cau[b]	Cdi[b]	Cef[b]	Cje[b]	Ckr[b]	Cpt[b]	Cur[b]	Cul[b]	Cva[b]	Cac1	Cac2	Cam	Cbo	Cge	Cgc1	Cgc2	Cli	Cma1	Cma2	Cpg	Cre	Cst	Ctu
CitA	41324296	145294130			25029461						340533901														
CitB	41324297	145294131			25029460						340533902														
MtrB	41324978	145294918	227832503	38233302	25027326	68536722	237786071	300685390	172040161	337290188	340535041	227076619	304568654	213952397	334564872	300533723	227182332	227093023	227077929	305660456	224946617	311304895	336102254	227197877	255299002
MtrA	41324977	145294917	227832502	38233301	25027325	68536723	237786072	300685389	172040160	337290187	340535042	227076618	304568653	213952343	334564873	300533724	227182331	227093024	227077928	305660570	224946616	311304896	336102255	227197876	255299003
PhoS	41326786	145296602	227834014	38234502	25029049	68535407	237784980	300686611	172041314	337291518	340533409	227077163	304567239		334564078	300534582	227183279	227090602	227079962	305660045	224945101	311304750	336100681	227197007	255297106
PhoR	41326787	145296603	227834015	38234503	25029050	68535406	237784979	300686612	172041315	337291519	340533408	227077162	304567240		334564077	300534581	227183278	227090601	227079961	305659950	224945100	311304749	336100682	227197006	255297105
CopS	41327150	145294165		38232698	25026835	68536517	237786492					227076977	304569272			300534212	227183034	227092686	227079416			311305614		227197243	255298015
CopR	41327151	145294164		38232699	25026834	68536518	237786491					227076978	304569273			300534213	227183035	227092687	227079417			311305613		227197242	255298016
HrrS	41327116	145296945	227834239	38234804	25029339			300686882		337291846		227077351	304566989			300534352	227180926	227090984	227080228	305659801	224945167	311304534		227197652	255298796
HrrA	41327115	145296944	227834238	38234803	25029338			300686881		337291845		227077352	304566988			300534353	227180927	227090985	227080229	305659839	224945168	311304535		227197653	255298797
CgtS8	41326188	145295914		38234860		68536239		300686928	172040516					213951479					227079388				336101787		
CgtR8	41326187	145295913		38234861		68536238		300686929	172040517					213951447					227079389				336101786		
CgtS1	41324504	145294405			25026790														227078880	305658374	224944376				
CgtR1	41324503	145294404			25026789														227078879	305658671	224947378				
CgtS2	41325099	145295045	227832654	38233452	25027505	68536617		300685533	172040226	337290327				213951269		300533392	227181626	227092289		305658576	224944281	311304393	336102197	227199235	255297550
CgtR2	41325098	145295044	227832653	38233451	25027504	68536618		300685532	172040225	337290326				213951290		300533393	227181625	227092288		305658477	224944282	311304394	c	227199236	255297551
CgtS4	41324637	145294520	227832116	38233004	25026980	68537005	237786476	300685129		337289922	340535607	227076699	304569017	213952347	334563886	300534035	227182596	227092860	227078179			311303785	336102491	227198299	255298758
CgtR4	41324638	145294521	227832117	38233005	25026981	68537004	237786475	300685130		337289923	340535606	227076700	304569018	213952570	334563885	300534034	227182597	227092859	227078180			311303784	336102490	227198300	255298759
CgtS5	41326843	145926660																							
CgtR5	41326842	145296659																							
CgtS6	41326941																								
CgtR6	41326942																								
CgtS7	41324836		227832317	38233206	25027175			300685304	172039772	337290099						300533863			227078005	305660541	224946672		336101290	227198096	
CgtR7	41324837	145294782	227832318	38233207	25027179			300685305	172039771	337290100						300533862			227078007	305660528	224946673		336101291	227198097	
CgtS10	41325174	145295106			25027577						340533252		304567074											227197728	
CgtR10	41325175	145295107			25027578						340533253		304567075											227197727	
cgR_2292		145296378																							
cgR_2299		145296385																							
cgR_0540		145294589							172040322							300532551									
cgR_0541		145294590							172040323							300532552									

[a] Cgl, *Corynebacterium glutamicum* ATCC 13032 (Ikeda and Nakagawa 2003; Kalinowski et al. 2003); CglR, *Corynebacterium glutamicum* R (Yukawa et al. 2007); Cau, *Corynebacterium aurimucosum* ATCC 700975 (Trost et al. 2010a); Cdi, *Corynebacterium diphtheriae* NCTC-13129 (Cerdeno-Tarraga et al. 2003); Cef, *Corynebacterium efficiens* YS-314 (Nishio et al. 2003); Cje, *Corynebacterium jeikeium* K411 (Tauch et al. 2005); Ckr, *Corynebacterium kroppenstedtii* DSM 44385 (Tauch et al. 2008a); Cpt, *Corynebacterium pseudotuberculosis* FRC41 (Trost et al. 2010b); Cur, *Corynebacterium urealyticum* DSM 7109 (Tauch et al. 2008b); Cul, *Corynebacterium ulcerans* BR-AD22 (Trost et al. 2011); Cva, *Corynebacterium variabile* DSM 44702 (Schröder et al. 2011); Cac1, *Corynebacterium accolens* ATCC 49725; Cac2, *Corynebacterium accolens* ATCC 49726; Cam, *Corynebacterium amycolatum* SK46; Cbo, *Corynebacterium bovis* DSM 20582; Cge, *Corynebacterium genitalum* ATCC 33030; Cgc1, *Corynebacterium glucuronolytium* ATCC 51866; Cgc2, *Corynebacterium glucuronolytium* ATCC 51867; Cli; *Corynebacterium lipophiloflavum* DSM 44291; Cma1, *Corynebacterium matruchotii* ATCC 14266; Cma2, *Corynebacterium matruchotii* ATCC 33806; Cpg, *Corynebacterium pseudogenitalium* ATCC 33035; Cre, *Corynebacterium resistens* DSM 45100; Cst, *Corynebacterium striatum* ATCC 6940; Ctu, *Corynebacterium tuberculostearicum* SK141.

[b] Completed and published genomes.

[c] Gene has not been annotated in the genome of *C. resistens* DSM45100, but re-analysis of the corresponding DNA region confirmed that it is present.
